# Preparation and in vitro evaluation of biological agents based on Zinc-laponite- curcumin incorporated in alginate hydrogel

**DOI:** 10.1186/s13036-023-00391-3

**Published:** 2023-11-24

**Authors:** Negar Karimi Hajishoreh, Mehdi Dadashpour, Abolfazl Akbarzadeh

**Affiliations:** 1 Health Research Center, Chamran Hospital, Tehran, Iran; 2 Department of Medical Biotechnology, Faculty of Medicine, Semnan University of Medical Sciences, Semnan, Iran; 3 Cancer Research Center, Semnan University of Medical sciences, Semnan, Iran; 4 Department of Medical Nanotechnology, Faculty of Advanced Medical Sciences, Tabriz University of Medical Sciences, Tabriz, Iran

**Keywords:** Laponite, Alginate, Hydrogel, Nanoparticulate systems

## Abstract

Due to their outstanding structures and properties, three-dimensional (3D) hydrogels and nanoparticles have been widely studied and indicated a very high potential for medical, therapeutic, and diagnostic applications. However, hydrogels and nanoparticles systems have particular drawbacks that limit their widespread applications. In recent years, the incorporation of nanostructured systems into hydrogel has been developed as a novel way for the formation of new biomaterials with various functions to solve biomedical challenges. In this study, alginate-loaded Zinc- laponite—curcumin (Zn/La/Cur) nanocomposites were fabricated via ionic cross-linking. The prepared nanocomposite hydrogels were characterized via FTIR and FE-SEM. Moreover, energy dispersive x-ray spectroscopy (EDX) was used to study the elements of the Zn/La/Cur nanocomposite. The NIH3T3 fibroblast cell line was utilized for the MTT assay to determine the cell viability of the fabricated alginate-loaded Zn/La/Cur nanocomposites. MTT results demonstrated that there was no evidence of toxicity in the samples. These outcomes suggest that applying Al/Zn/La/Cur nanocomposite as a biological agent could be a novel tissue engineering strategy for treating soft tissue disorders.

**Graphical Abstract:**

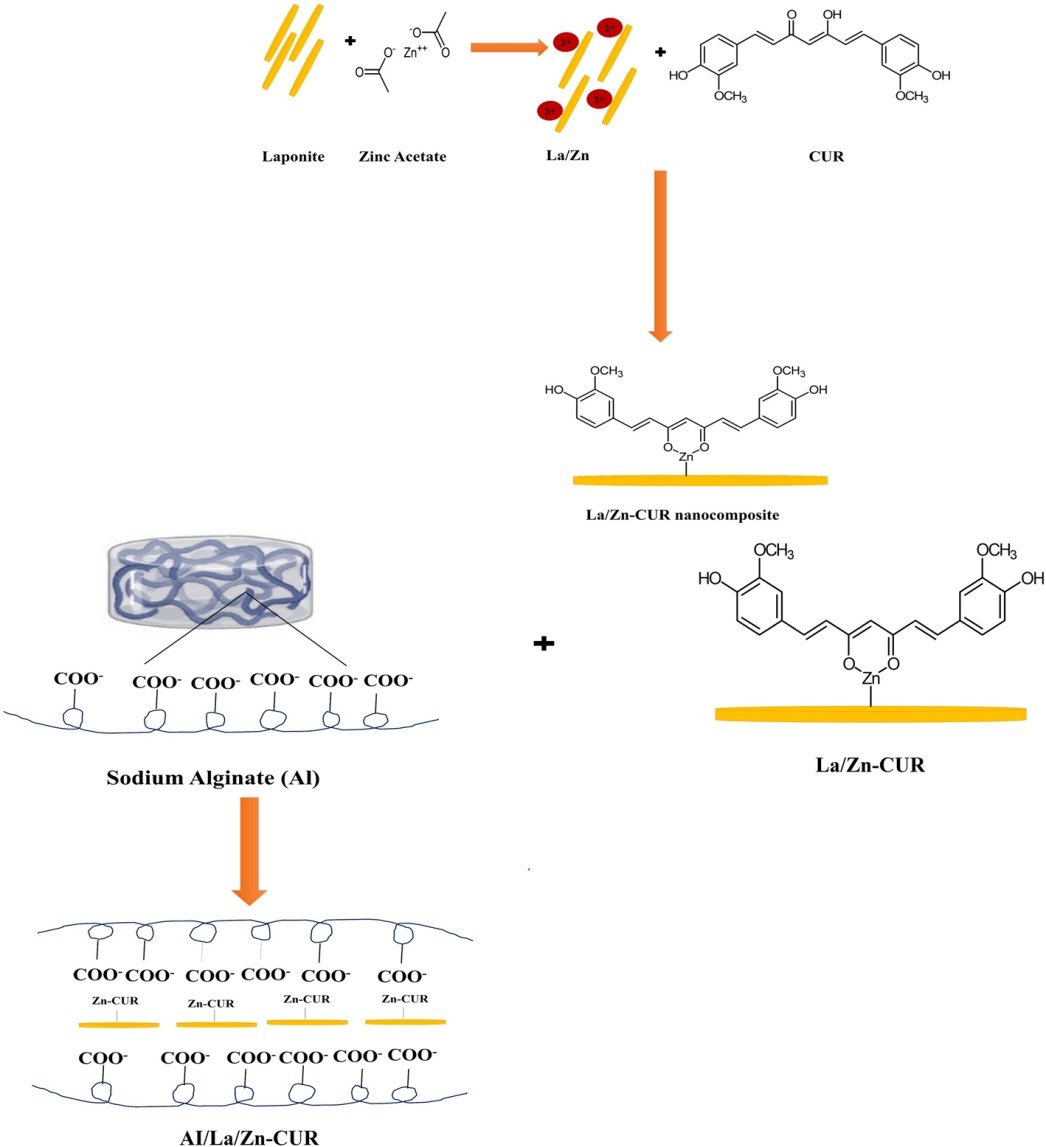

## Introduction

Hydrogels, as extremely hydrophilic macromolecular networks, are attractive candidates for tissue engineering applications and regenerative medicine. The principal goal of tissue engineering is to prompt recovery of damaged tissue with poor scarring and maximum regeneration [1]. Without hesitation, the skin is the first line of body defense against external environmental factors and compared to other organs, is more exposed to risks such as burns, cuts, and stretching. Based on the rate of skin damage, the methods of treatment are different. Therefore, using a technique that can accelerate the healing process of skin injuries could lead to an increase in the survival of patients [2]. Wound healing, including regular and complex procedures. When skin damage happens, the wound healing process begins immediately, but up to complete epithelialization, patients remain assailable to multiple penetration microbial infections. Recently, tissue engineering commends novel approaches to control the rate of mortality by increasing the quality rate of the healing process in skin disorders.

Wound dressing based on hydrogels due to having unique characteristics are promising materials in wound treatment [3]. According to investigations, wound dressings have some common properties not toxic, being able to absorb secretions from the surface of the wound, helping to exchange gases and moisture, creating proper humidity in the contact area, preventing the penetration of microorganisms into the wound, easily separated from the surface of the wound. In these circumstances, hydrogel dressings are not only capable mentioned above, but they could also remove the wound exudates encouraging fibroblast proliferation and keratinocyte migration [4] Natural hydrogel with unique physical and biological properties such as biocompatibility, adjustable mechanical properties, biodegradability, and in situ crosslinking abilities are considered candidates to engineer epidermis [5]. Alternatively, critical properties of hydrogels, such as low mechanical strength and extensibility limit their use for wound healing applications. Therefore, the study about the effect of incorporating polymeric, ceramic, and metallic nanoparticles into the hydrogel has been considered to overcome these restrictions [6]. In a recent study polysaccharide-based pH-sensitive hybrid hydrogel was designed and used to for delivery of amoxicillin and ornidazole as a sustained-release matrix [7].

In this regard, laponite (La) has attracted significant attention because of its ability to support cell adhesion, proliferation, differentiation, DNA hemostatic properties. Moreover, the presence of both positive and negative charges on the surface of layered silicate nanostructure results in an exclusive anisotropic structure at the nano and microscales [8, 9]. Besides, Alginate is an anionic, hydrophilic, biocompatible, and biodegradable polymer at normal pH. Sodium alginate can form scaffolds within a relatively short period and can easily be manipulated to regulate the level of porosity [10].

Newly, biomedical science tries to inventnovel, low-cost, safe molecules that may be used in the handling of inflammatory, neoplastic, and infectious diseases. Curcumin (CUR), the primary natural polyphenol found in the root of Curcuma longa, is a bioactive component with proven antioxidant, and anti-inflammatory potentials [11]. Moreover, according to the last investigations, CUR can interact with the main molecules involved in skin regeneration. Also, numerous reports have proved that CUR possessed intense activities on various cancers, especially breast cancer cells, by arresting different cell cycle stages and inducing apoptosis [12].

The main objective of the current work was to develop a novel nanohybrid hydrogel composed of alginate-loaded Zn/La/Cur with adjustable physical and biological properties to support NIH3T3 fibroblast cells with reconstructive capacity in vitro.

## Materials and methods

### Materials

Laponite (RD, containing 59.5% SiO_2_, 27.5% MgO, 0.8% Li_2_O, and 2.8% Na_2_O,) was purchased from BYK Additives & Instruments, Germany. Sodium alginate (ALG, Mw = 1.93 × 105 g/mol) was purchased from Aladdin Reagent Company (Shanghai, China). Curcumin, and thiazolyl blue tetrazolium bromide (MTT) were obtained from Sigma–Aldrich. Zinc acetate and dimethyl sulfoxide (DMSO) were supplied by Merck. Fetal bovine serum (FBS), Phosphate buffered saline (PBS), trypsin–EDTA, Penicillin–Streptomycin (Pen/strep), and RPMI 1640 medium were obtained from Gibco.

#### Fabrication of Zn/La/Cur nanocomposite

To prepare Zn/La/Cur nanocomposite, Firstly, 0.02 mg laponite powder was dispersed in deionized water and sonicated for 20 min. Consequently, Zinc acetate (150 mg) was added to the laponite suspension and stirred for 2 h. Curcumin (3 mg) was added to the Zn/La suspension,and the mixture was vigorously stirred for 2 h. Then, the resulting suspension was centrifuged and washed with deionized water to remove the residual agents. Finally, the as-obtained product was dried via the freeze-dryer method.

#### Preparation of alginate-loaded Zn/La/Cur nanocomposite

**Fig. 1:**
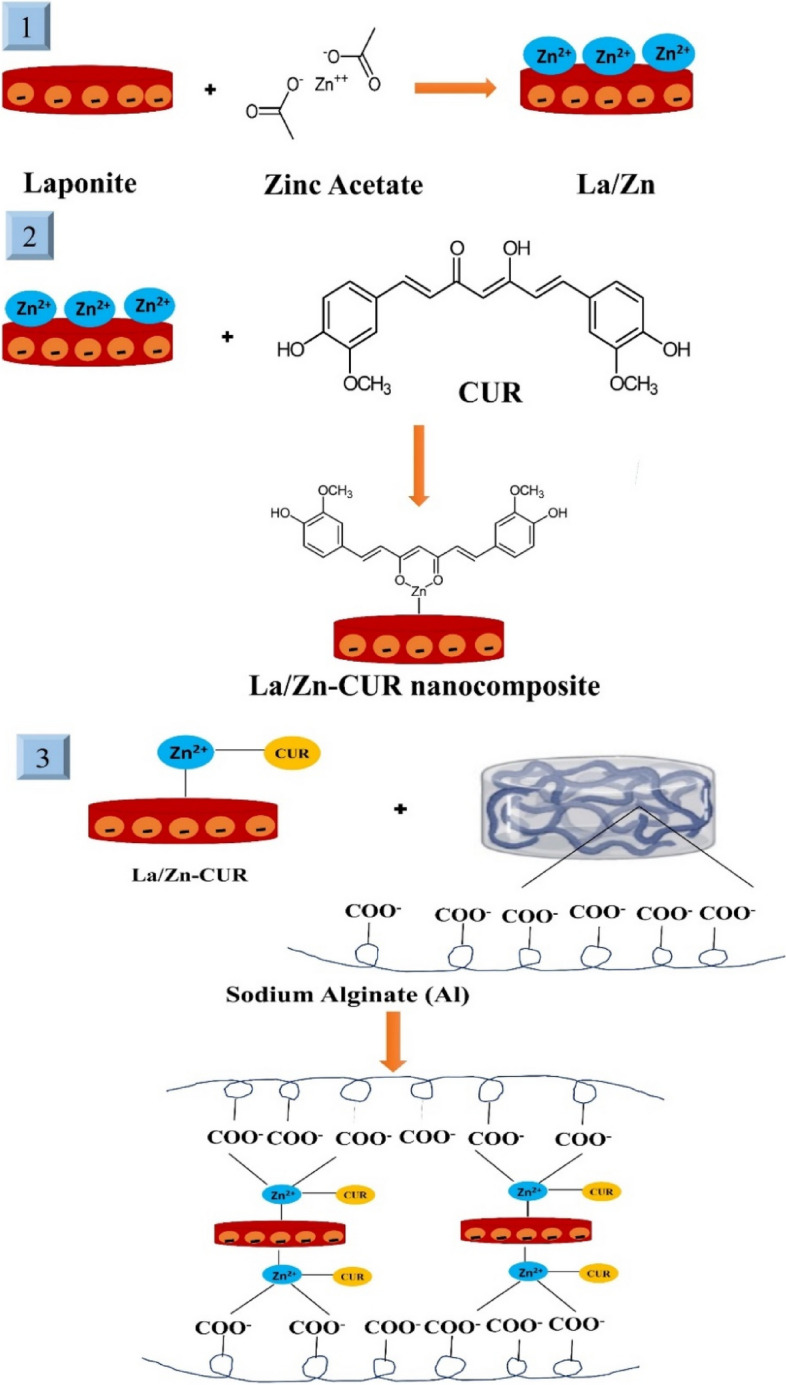
A schematic for hydrogel preparation using the chemical structure of alginate. The crosslinking reaction and interaction with the Zinc- laponite – curcumin

The alginate-loaded Zn/La/Cur nanocomposite (Al/Zn/La/Cur nanocomposite) were fabricated according to the previous study with some modifications [13]. For this purpose, alginate solution (1% w/v) was prepared via dissolving in deionized water overnight. The various amounts of Zn/La/Cur nanocomposite dispersion (1 mL, 0.5 mL, and 0.25ml) were introduced separately to the alginate solution (2 mL); subsequently, the samples were injected in the 2 wt.% CaCl_2_ bain marie for 5 min to ionically crosslink alginate units. Finally, the obtained Al/Zn/La/Cur nanocomposite were immersed in deionized water to remove the excessive CaCl_2_. Figure 1 showed the schematic of hydrogel preparation using the chemical structure of alginate.

## Characterizations

### Hydrogel characterizations

For investigation of the chemical structure of the samples, Fourier transform infrared spectroscopy (FTIR) was employed. The FTIR spectrum of each sample was recorded by the FTIR spectrometer (Bruker-Tensor 27) in the range of 400–4500 cm^_1^. The particle size distribution of laponite and Zn/La/Cur nanocomposite in suspensions was performed at the temperature of 25ºC by dynamic light scattering (DLS, Malvern Instruments, Ver. 7.11) measurement.

The La/Zn-CUR nanocomposite surface morphology was conducted using field emission scanning electron microscopy (FE-SEM, MIRA3, Tuscan). For FE-SEM imaging, the freeze-dried samples were sputter coated with a thin layer of gold. Also, energy dispersive X-ray spectroscopy (EDX) was used to study the elements of the Zn/La/Cur nanocomposite. X-ray diffraction (XRD. Ital structure, MPD 3000) was used to analyze prepared samples with a 2θ range from 10° to 80° and a step size of 0.001.

### Rheological study

Rheological measurements were done using a Modular Compact rheometer (MCR 300, Anton Paar GmbH, Austria) at 25 °C, with a Cone–Plate measuring geometry, CP 25–2, having radius of 12.5 mm. Steady shear viscosity measurements were performed at various shear rates ranging from 0.005 s^−1^ to 100 s^−1^.

Rheological measurements were performed using rheometer Gemini 200 (Molvern Instruments, UK). Cone-plate geometry with 40 mm disk diameter was used in the experiments. The upper plate was having cone angle of 4 degrees and there was 150 μm gap between the upper and lower plate. The hot.

### In vitro cell bioavailability assay

The MTT assay was used to investigate the cytocompatibility of the prepared hydrogels in the NIH3T3 fibroblast cell line [14]. The cells were cultured in RPMI 1640 medium containing 10% (v/v) FBS and 1% (v/v) Pen/Strep. For biocompatibility characterizations, the fabricated structures were cut into circular shapes and, after UV radiation, placed in the 96-well plate. Afterward, Al/Zn/La/Cur nanocomposite were washed using sterilized PBS 3 times. Accordingly, NIH3T3 cells with a density of 5.0 × 10^3^ cells were seeded onto the wells and incubated at 5% CO_2_ and 37 ºC. After three days, the culture medium in the wells was removed and replaced with MTT solution (3 mg/mL in PBS) and incubated for 4 h. Then, the MTT reagent was discarded, and DMSO (200 µL) was added to each well to dissolve formazan crystals. Finally, the absorbance of the samples was measured by a Microplate Reader (Awareness Technology).

NIH3T3 fibroblast cells were seeded according to the previous section that evaluated the cell attachment on the fabricated Al/Zn/La/Cur nanocomposite. After 3 days of seeding, the medium of each well was removed, the cell seeded Al/Zn/La/Cur nanocomposite were rinsed with PBS, and cells were fixed with glutaraldehyde (4% v/ v in PBS) for 20 min. For dehydration of samples, the series of ethanol solutions (50%, 60%, 70%, 80%, 90%, and 100%) were applied. Finally, the images of cells on the sample surfaces were recorded with FE-SEM.

## Statistical analysis

Statistical analysis was performed by one-way analysis of variance (ANOVA) via Graph PAD Prism software. All the experimental results were expressed as means ± standard deviations, and *p*-value < 0.05 was described as the statistically significance.

## Results

### Investigation of the chemical structure of fabricated hydrogel by FTIR

**Fig. 2:**
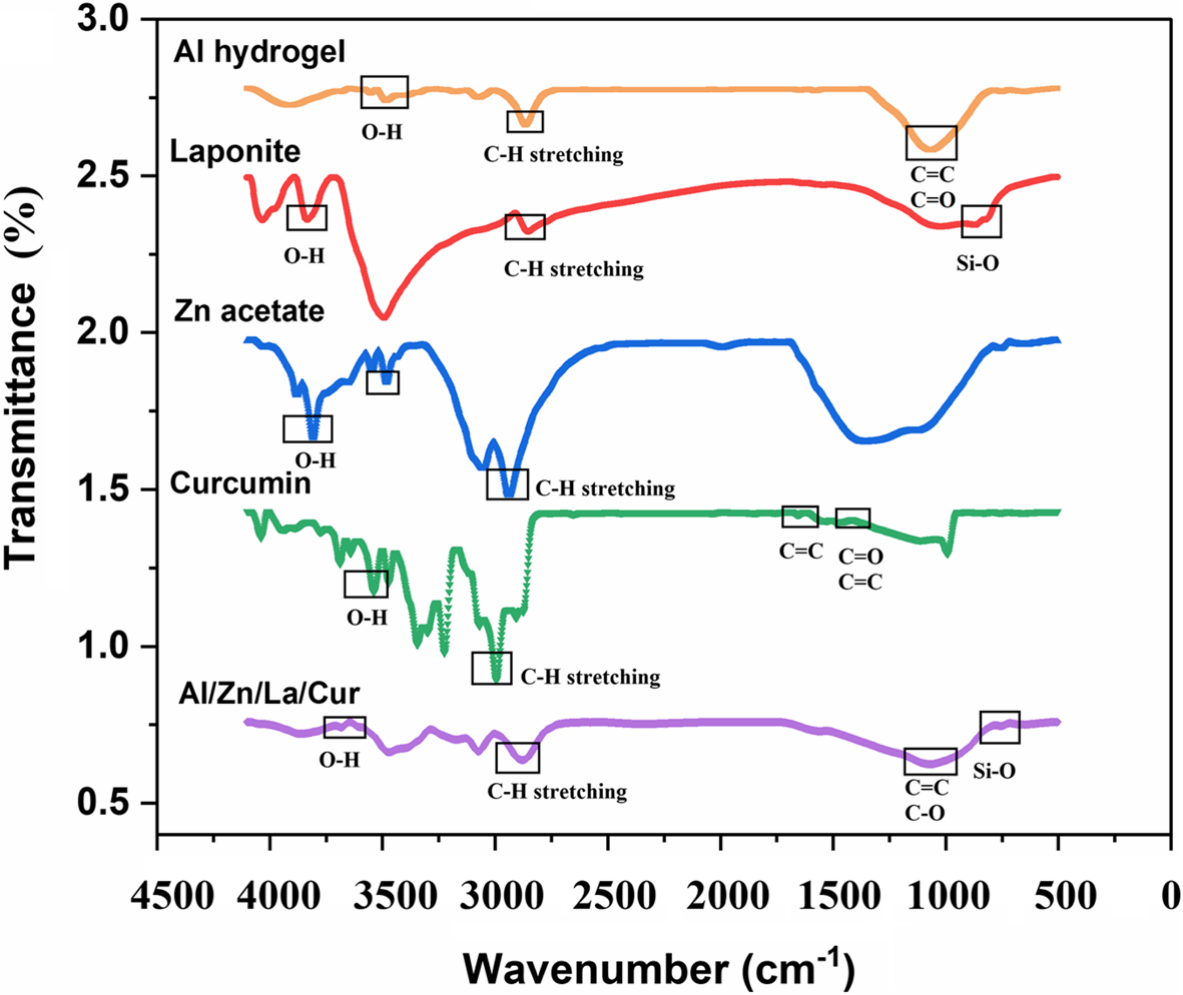
FT-IR spectra of pure Al, La, Zn acetate, Curcumin, and Al/Zn/La/Cur hydrogel

Chemical structure of Al/La/Zn-CUR nanohybride was confirmed using FT-IR analysis. As represented in Fig. 2, pure alginate demonstrates the characteristic absorption bands at 1250 cm^−1 1^, which are attributed to asymmetric and symmetric stretching vibrations of carboxylate salt ion groups, respectively. Moreover, the peaks around 3208 cm^−1^ and 2925 cm^−1^ are associated with OH stretching and stretching vibrations of aliphatic C-H, respectively [15, 16]. Cur exhibits a sharp peak at 3543 cm ^−1^ (phenolic O–H stretching vibration), 1730 cm ^−1^(aromatic moiety C = C stretching), 1610 cm^−1^ (benzene ring stretching vibrations), 1501 cm^−1^ (C = O and C = C vibrations), 1420 cm ^−1^ (olefinic C-H bending vibrations), 1011 cm ^−1^ (C–O–C stretching vibrations) [17, 18]. The characteristic bands for laponite were observed at 3839 and 2700 cm^−1^ (OH-stretching of the silicate layer) and 732 cm^−1^ (Si–O – bending). To develop nanohybrid hydrogels, laponite nanoparticles consisting of plate-like particles with an average size of 75.5 nm were integrated into the matrix. FT-IR spectrum of laponite, and characteristic bands showed the presence of distinctive absorption bands, which were similarly reported in prior research. FT-IR spectrum of Al/La/Zn-CUR nanohybride consisted of the characteristic bands of laponite, alginate, and Cur with some differences.

### Dynamic light scattering (DLS) and X-ray spectroscopy (EDX)measurements

**Fig. 3:**
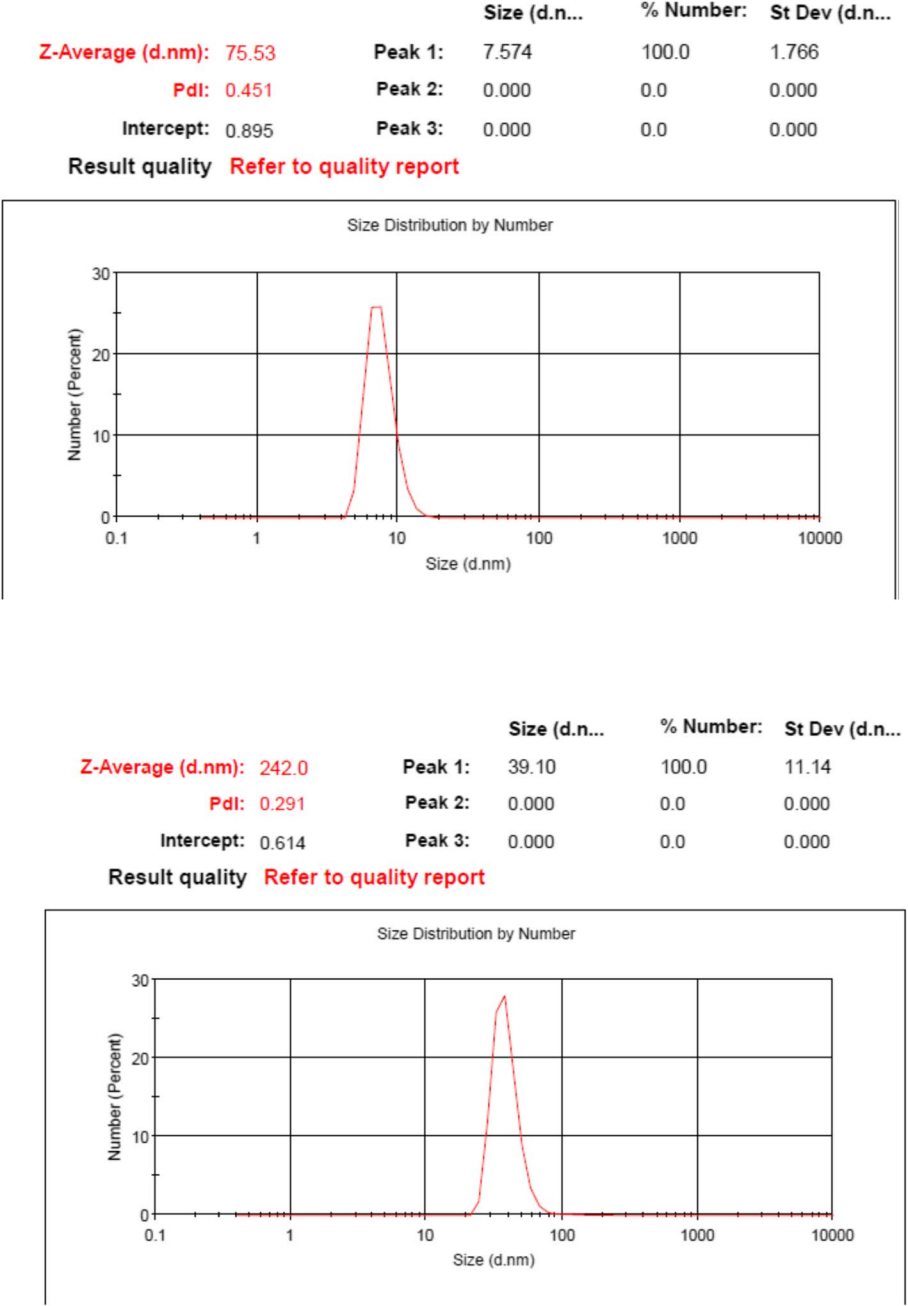
Particle size distribution from the dynamic light scattering (DLS) of Laponite and Zn/La/Cur nanocomposite

**Fig. 4:**
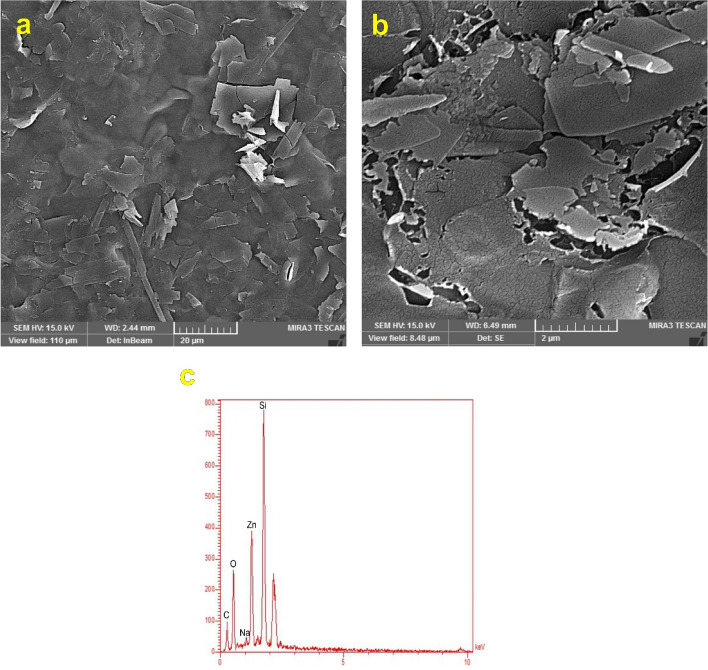
**A** FE-SEM images of Laponite and Zn/La/Cur nanocomposites. **B** Energy-dispersive X-ray spectroscopy of Zn/La/Cur nanocomposite

Also, DLS measurement was used to assess the diameter and size distribution of nanoparticles. As is observed in Fig 3, laponite displayed an average particle size of 75.5 nm with uniform size distribution and a PDI of 0.451. The Al/La/Zn-CUR nanocomposite represented a large size with a mean diameter of 242 nm and a narrower particle size distribution (PDI= 0.291). X-ray spectroscopy (EDX) of Al/La/Zn-CUR nanohybride confirmed the presence of laponite, Zn, and CUR within Al/La/Zn-CUR nanohybride (Fig 4c).

### Morphological evaluation via FE-SEM

#### Rheological study

The FE-SEM image of the alginate hydrogel and Al/La/Zn-CUR nanocomposite exhibited a microporous structure (Fig. 4). These pores enable the scaffolds to transport nutrient and exit waste products in the wound site. Moreover, nanocomposite scaffolds promoted NIH3T3 fibroblast cell adhesion and proliferation.

**Fig. 5:**
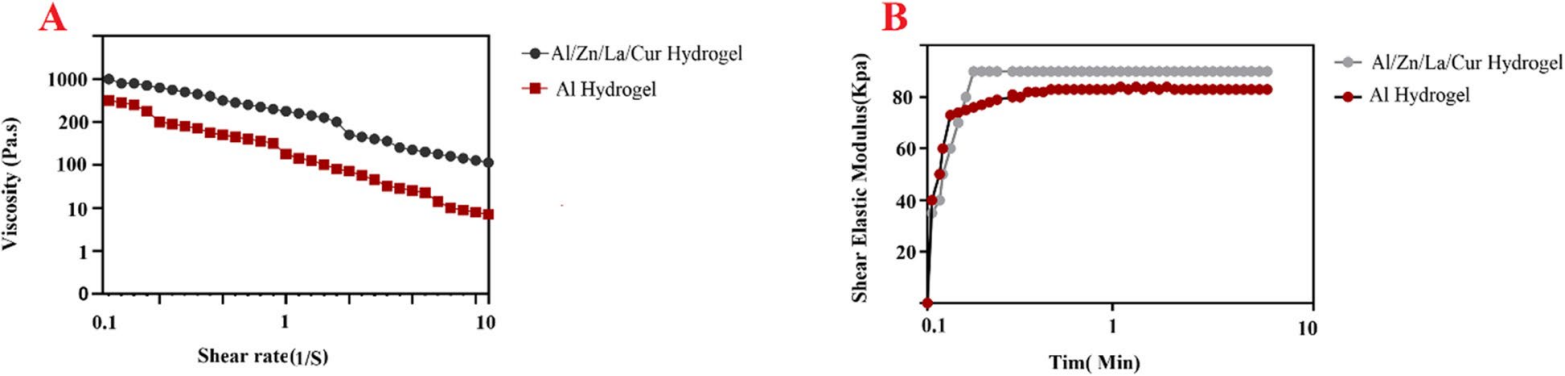
Rheological properties of the hydrogels. **A** Steady shear viscosity measurements of SA with and without La/Zn-CUR. **B** Gelation time

The flow properties of non-crosslinked hydrogels were the key factor for cell viability during printing process. The plots of viscosity and shear stress against the shear rate were obtained for the prepared Al/La/Zn-CUR hydrogels. At 25 °C, viscosity of the hydrogels was decreased with increasing shear rate. Figure 5 displays the rheological behavior of as prepared precursors. As observed, the addition of La/Zn-CUR increased the viscosity of precursor while changing its behavior from Newtonian to thixotropic.

### MTT assay

**Fig. 6:**
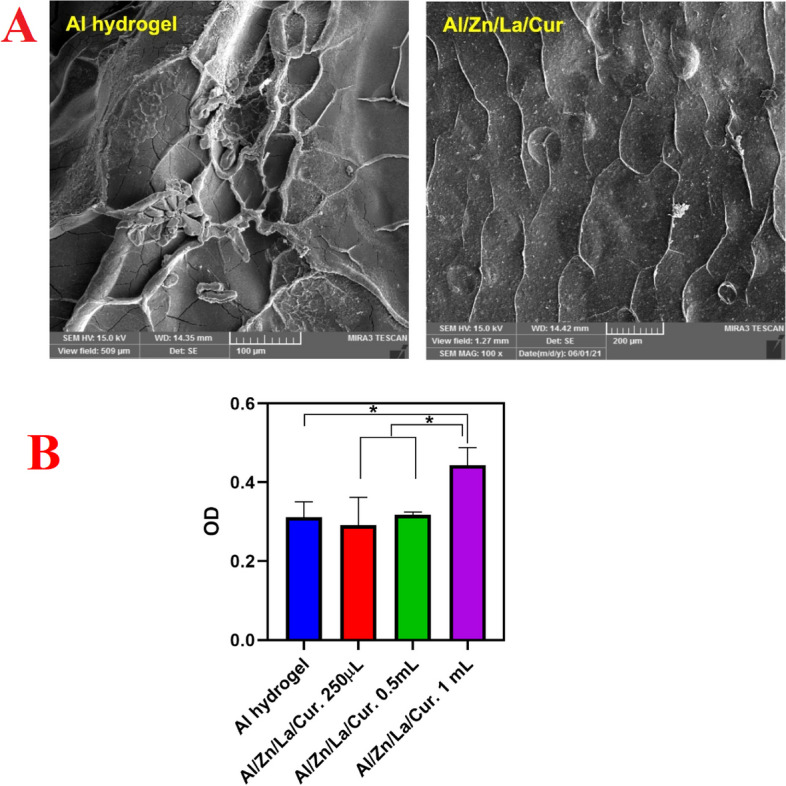
Cellular studies. **A** FE-SEM images of the cell attachment on the nanocomposites after 7 day. **B** In vitro biocompatibility of NIH3T3 fibroblast cell line on the nanocomposites over 7 day using MTT assay

**Fig. 7:**
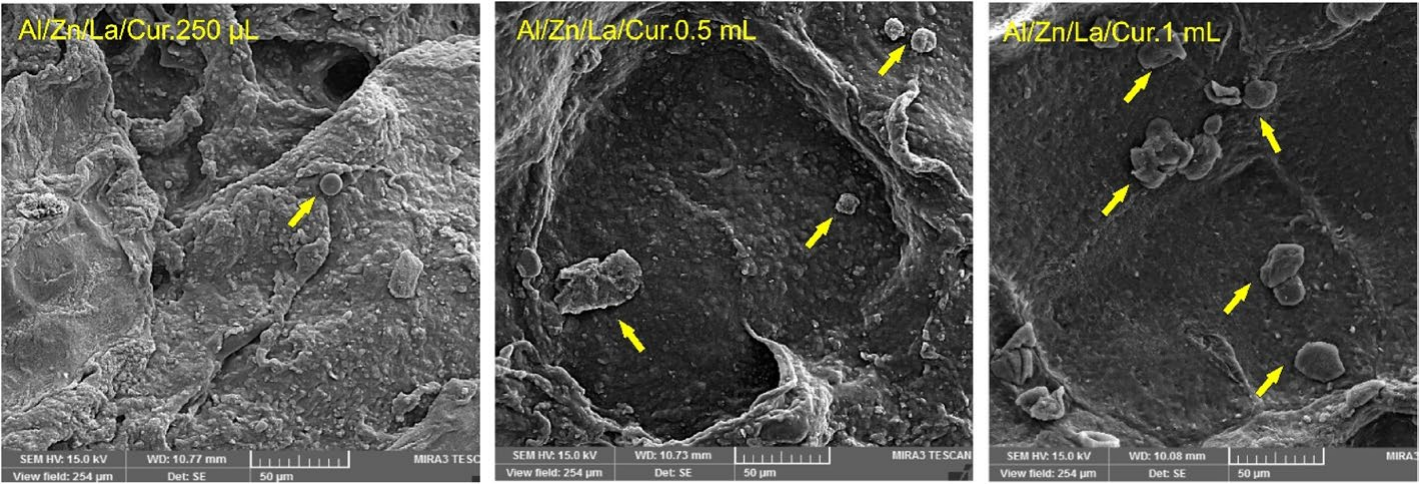
Cellular studies. FE-SEM micrographs of seeded fibroblasts on nanocomposites after 7 days of culture (scale bar: 50 µm)

The viability and proliferation of NIH3T3 fibroblast cells on nanocomposite scaffolds via MTT assay was investigated after 7 days of cell seeding in Fig. 6B. The obtained results indicated cell viability and proliferation enhanced with increasing the concentration of CUR. Outcomes showed that Al/La/Zn-CUR nanohybride could be a novel strategy for treating skin tissue engineering at the optimal concentration of CUR which will give appropriate swelling with enhanced biocompatibility as well. As illustrated in Fig. 7, after 7 days of seeding, the cells attach and spread on the surface of all matrices. The cell evaluation results have approved that the biocompatible scaffold based on Al/La/Zn-CUR nanocomposite could improve cell–cell interaction by providing ECM, mimicking the native microenvironment.

## Discussion

The numerous preparation techniques adopted are physical cross-linking, chemical cross-linking grafting polymerisation, and radiation cross-linking [19]. Based on the kinds of cross-link junctions, hydrogels can be classified into two groups: the chemically cross-linked and the physically cross-linked. Chemically cross-linked gels have stable junctions, in which covalent bonds are present between different polymer chains, thus leading to outstanding mechanical strength. Such modifications can increase the mechanical properties and viscoelasticity for uses in biomedical and pharmaceutical arenas [20]. Cross-linking is a stabilization procedure in polymer chemistry that resulting in the multi-dimensional extension of polymeric chains, leads to network structures. By crosslinking, hydrogels are formed into stable structures that differ from their raw materials. Though chemical cross-linking is an extremely inventive technique for the formation of hydrogels, the cross-linkers used in hydrogel preparation should be extracted from the hydrogels before use, due to their reported toxicity, whereas, in physically cross-linked gels, dissolution is prohibited by physical interactions, such as ionic interactions, hydrogen bonds or hydrophobic interactions [21, 22].

Injectable gels have numerous advantages over preformed implants, including their non-invasive introduction in vivo and the capability to be used to homogeneously macromolecules and encapsulate cells. The elastic characteristics of gel can be improved by variable the amount of concentration. Mechanical, biochemical and rheological properties of hydrogels are powerfully connected to their chemical composition, the way hydrogel is polymerized and density of cross linkers [23].

Despite considerable improvement in therapeutic approaches, skin disorders remain a challenging discussion. Over the past decade, research on hydrogel-based material for prepose of skin regeneration has become a promising approach [24].The incorporation of nanoparticles into the hydrogel and transfer in the damaging side as a wound dressing has shown acceptable results including, improving absorption of wound exudates, decreasing infections and adverse allergic effects influences improving wound regeneration [25]. Natural hydrogel polymers exhibit biological activities such as cell recruiting, improving neo-vasculature, and modulation of the inflammatory microenvironment [26, 27]. Among the natural hydrogels-based materials for skin tissue engineering, alginate has been considered a biocompatible and hemostatic polymer [27]. Due tothe poor mechanical characteristics of alginate, we hypothesized that the incorporation of Zinc-dopped laponite nanoparticles in alginate could improve mechanical properties and facilitate the fabrication of regular microporous structures that are suitable for cell nutrient transferring. Moreover, Curcumin (CUR) is documented as a harmless composite by the Food and Drug Administration (FDA) and, numerous preclinical and clinical studies assessed in this field. The wound healing process is a dynamic and complex process. During the inflammatory phase of wound healing CUR could regulate inflammation via regulating main signaling pathways and reducing concentration target molecules like TNF-and IL-1 and fibroblast recruiting, releasing protease and removing the rate of reactive oxygen species (ROS) as well [28]. In the proliferation stage, CUR also has a critical role in the differentiation of fibroblast and collagen synthesis, decreasing the level of the number of membrane matrix metallo-proteinases (MMPs) [29]. We hypothesized that the incorporation of Zinc-dopped laponite containing curcumin within alginate (Al/La/Zn-CUR) could promote the viability of the cell-encapsulated alginate, which in turn could fabricate the novel structure for maximum transporting nutrients and exit waste products in the wound site.

Our results were in line with the published literature data, emphasizing that the incorporation of Zinc-dopped laponite containing CUR within alginate could be beneficial in improving viability.

As mentioned above, due to the aforementioned fantastic alginate assets, hydrogels as promising constituents are highly suitable for diverse applications, particularly for diagnostic and therapeutic manners in biomedical areas. They not only can assist as a carrier to load and transfer remedy or protein to tissues [5, 30] but also can act as scaffold to replace damaged tissues and organs, helping as wound dressings, barriers, or adhesives membrane between material and tissue surfaces [31, 32]. Recently, tissue engineering technology has assisted in fabricate various commercial wound dressings based on natural and synthetic hydrogels [33]. Wound dressings based on biological nanocomposites have helped to increase wound healing managements [34, 35]. In a study that was prepared from a wound dressing based on zinc oxide- alginate, antibacterial outcomes, they had somewhat advanced antibacterial activities against *S. aureus* than E. coli.

Consequently, sodium alginate (SA)-Zinc oxide (ZnO) nanoparticle has the potential to be used as a wound healing material in biomedical applications [36]. Laponite is a nanomaterial with a disc-like crystal structure that has a large surface area compared to its volume. Duo to it exhibit unique properties like low toxicity when interacting with the body’s microenvironment is widely considered in regenerative medicine [37, 38]. Moreover, in the tissue engineering field, Laponite could suppress the immunological body responses and stimulate differentiation and proliferation of host cells, when applied as a vector. Also, this nanoparticle, when incorporated in hydrogel /scaffolds structure could increase mechanical resistance as well [39, 40].

## Conclusions

This study aimed to fabricate novel nanohybrid hydrogels based on Al/Zn/La/Cur and investigate the effects of Curcumin concentration on the physical and biological properties of the hydrogels. Results confirmed that the Al/Zn/La/Cur nanocomposite has no toxic effects on human fibroblast skin and NIH3T3 cells. However, our results indicated that developed Al/Zn/La/Cur nanocomposites could be a promising achievement for wound healing. However, more experiments are still needed to recognize the safety and efficiency of this hydrogel for clinical application.

## Data Availability

Sharing not applicable to this article as no data-sets were generated. Data analysis in the current study was performed using publicly available datasets.

## References

[1] Sabry NM (2018). Interaction between nano silver and bacteria: modeling approach. Biointerface Res Appl Chemist.

[2] Stoica AE, Chircov C, Grumezescu AM (2020). Hydrogel dressings for the treatment of burn wounds: an up-to-date overview. Materials.

[3] Kaur P, Gondil VS, Chhibber S (2019). A novel wound dressing consisting of PVA-SA hybrid hydrogel membrane for topical delivery of bacteriophages and antibiotics. Int J Pharm.

[4] Landis, S., et al., Infections in chronic wounds. Chronic wound care: a clinical source book for healthcare professionals. 4th ed. Malvern, PA: HMP Communications, 2007: p. 299–321.

[5] Slaughter BV, Khurshid SS, Fisher OZ, Khademhosseini A, Peppas NA (2009). Hydrogels in regenerative medicine. Adv Mater.

[6] Hoare TR, Kohane DS (2008). Hydrogels in drug delivery: Progress and challenges. Polymer.

[7] Das D, Roy A, Pal S (2023). A polysaccharide-based ph-sensitive hybrid hydrogel as a sustained release matrix for antimicrobial drugs. ACS Applied Polymer Materials.

[8] Zhang F (2020). Selective oxidation of H2S over Fe supported on Zr-intercalated Laponite clay mesoporous composite catalysts at low temperature. Catal Today.

[9] Kiaee G (2022). Laponite-based nanomaterials for drug delivery. Adv Healthcare Mater.

[10] Goh CH, Heng PWS, Chan LW (2012). Cross-linker and non-gelling Na+ effects on multi-functional alginate dressings. Carbohyd Polym.

[11] McClements DJ, Li F, Xiao H (2015). The nutraceutical bioavailability classification scheme: classifying nutraceuticals according to factors limiting their oral bioavailability. Annu Rev Food Sci Technol.

[12] Vollono L (2019). Potential of curcumin in skin disorders. Nutrients.

[13] Asadi N (2023). Preparation and characterization of propolis reinforced eggshell membrane/GelMA composite hydrogel for biomedical applications. BMC Biotechnol.

[14] Javan ES (2022). Development of a magnetic nanostructure for co-delivery of metformin and silibinin on growth of lung cancer cells: possible action through leptin gene and its receptor regulation. Asian Pacific J Cancer Prevent.

[15] Pereira R (2011). Preparation and characterization of films based on alginate and aloe vera. Int J Polym Anal Charact.

[16] Saarai A (2013). On the development and characterisation of crosslinked sodium alginate/gelatine hydrogels. J Mech Behav Biomed Mater.

[17] Firouzi Amandi A (2023). Enhanced anti-cancer effect of artemisinin-and curcumin-loaded niosomal nanoparticles against human colon cancer cells. Med Oncol.

[18] Amirsaadat S (2023). Potential anti-proliferative effect of nano-formulated curcumin through modulating micro RNA-132, Cyclin D1, and hTERT genes expression in breast cancer cell lines. J Cluster Sci.

[19] Lu L (2018). The formation mechanism of hydrogels. Curr Stem Cell Res Ther.

[20] Zhang W (2020). Catechol-functionalized hydrogels: biomimetic design, adhesion mechanism, and biomedical applications. Chem Soc Rev.

[21] Asadi N (2023). Preparation and characterization of propolis reinforced eggshell membrane/GelMA composite hydrogel for biomedical applications. BMC Biotechnol.

[22] Ghasemiyeh P, Mohammadi-Samani S (2019). Hydrogels as drug delivery systems; pros and cons. Trends Pharmaceu Sci.

[23] Rana P, Ganarajan G, Kothiyal P (2015). Review on preparation and properties hydrogel formulation. WJPPS.

[24] Karimi Hajishoreh N (2020). Reduced graphene oxide facilitates biocompatibility of alginate for cardiac repair. J Bioact Compat Polym.

[25] Karimi Hajishoreh N (2022). Left ventricular geometry and angiogenesis improvement in rat chronic ischemic cardiomyopathy following injection of encapsulated mesenchymal stem cells. Cell J.

[26] Balakrishnan B (2005). Evaluation of an in situ forming hydrogel wound dressing based on oxidized alginate and gelatin. Biomaterials.

[27] Ye Z (2011). Myocardial regeneration: roles of stem cells and hydrogels. Adv Drug Deliv Rev.

[28] Velnar T, Bailey T, Smrkolj V (2009). The wound healing process: an overview of the cellular and molecular mechanisms. J Int Med Res.

[29] Barchitta M (2019). Nutrition and wound healing: An overview focusing on the beneficial effects of curcumin. Int J Mol Sci.

[30] Zhao F (2015). Composites of polymer hydrogels and nanoparticulate systems for biomedical and pharmaceutical applications. Nanomaterials.

[31] Caló E, Khutoryanskiy VV (2015). Biomedical applications of hydrogels: A review of patents and commercial products. Eur Polymer J.

[32] Peppas NA (2006). Hydrogels in biology and medicine: from molecular principles to bionanotechnology. Adv Mater.

[33] Nabizadeh Z (2022). Micro-and nanotechnology in biomedical engineering for cartilage tissue regeneration in osteoarthritis. Beilstein J Nanotechnol.

[34] Silva R (2012). Wound-healing evaluation of entrapped active agents into protein microspheres over cellulosic gauzes. Biotechnol J.

[35] Deldar Y (2018). An in vitro examination of the antioxidant, cytoprotective and anti-inflammatory properties of chrysin-loaded nanofibrous mats for potential wound healing applications. Artificial Cells Nanomed Biotechnol.

[36] Mohandas A (2015). Exploration of alginate hydrogel/nano zinc oxide composite bandages for infected wounds. Int J Nanomed.

[37] Chiu C-W (2014). Intercalation strategies in clay/polymer hybrids. Prog Polym Sci.

[38] Tomás H, Alves CS, Rodrigues J (2018). Laponite®: a key nanoplatform for biomedical applications? Nanomedicine: Nanotechnology. Biology Med.

[39] Dawson JI, Oreffo RO (2013). Clay: new opportunities for tissue regeneration and biomaterial design. Adv Mater.

[40] Nejati K (2022). Nanoparticle-based drug delivery systems to overcome gastric cancer drug resistance. J Drug Delivery Sci Technol.

